# Isiris: A Novel Method of Removing Foreign Bodies from the Lower Urinary Tract to Avoid Unnecessary Hospitalization and Anesthesia

**DOI:** 10.1089/cren.2016.0086

**Published:** 2016-08-01

**Authors:** Peter Mark Smith, Aman Harbias, Richard Robinson, Anne Palmer, Benjamin Robin Grey

**Affiliations:** Department of Urology, Central Manchester Hospitals Foundation Trust, Manchester, United Kingdom.

**Keywords:** Isiris, foreign body, polyembolokoilamania, genitourinary

## Abstract

***Background:*** Polyembolokoilamania refers to the practice of inserting foreign bodies (FBs) into natural orifices. A FB within the urethra is a relatively rare phenomenon with 646 cases recorded last year in the United Kingdom. Management of these patients presents technical challenges and complexities because of underlying psychiatric disorders that are often associated. This case illustrates a novel way of removing FBs from the genitourinary tract, requiring less resources, preventing hospital admission, and attempts to break the cycle of behavior, leading to recurrent attendance with polyembolokoilamania.

***Case Presentation:*** A 38-year-old Caucasian male prisoner, with psychiatric history presented to the emergency department (ED) with a history of inserting FBs into his urethra on 12 different occasions over a 6-week period. Of these 12 attendances, 3 resulted in admission and 2 required emergency intervention in theater under general anesthesia. After the third attendance in 5 days, it was decided to use Isiris™, a single-use flexible cystoscopy device with a built-in ureteral stent grasper, to remove the FBs and check the integrity of the urethra. The procedure was performed within the ED, without the need for admission to a ward bed or general anesthesia. Furthermore, only two members of staff were required to remove all of the urethral FBs.

***Conclusion:*** Isiris, although marketed as a stent removal device, enabled us to remove all the patient's FBs in one procedure. Isiris is an easy to use device, similar to a flexible cystoscope, that a specialist nurse or resident would be familiar using. It allows efficient and safe removal of lower urinary tract FBs, even out of hours. It requires minimal staffing support and can be done in the ED. It has the potential to reduce associated sequela of urethral polyembolokoilamania, saving resources while preserving the availability of the emergency theater.

## Introduction

Polyembolokoilamania refers to the practice of inserting foreign bodies (FBs) into natural orifices. FBs in the urethra are a relatively rare phenomenon. Of the 5.6 million emergency hospital admissions recorded in England in a 1-year period between April 2014 and March 2015, 646 were because of FBs in the genitourinary (GU) tract with 89 resulting procedures recorded (6 open, 83 endoscopic) for their removal.^1^

A large number of patients who insert FBs into their urethra may do so on multiple occasions, thus the management of these patients poses challenges of both a psychiatric and technical nature. When theater time and resources are required to remove FBs, the costs to the healthcare provider organization markedly increase. This case illustrates a novel method of removing FBs from the lower GU tract, which has the potential to save resources, prevent hospital admissions, and to break the cycle of behavior that leads to recurrent polyembolokoilamania.

## Case Report

**FIG. 1.**
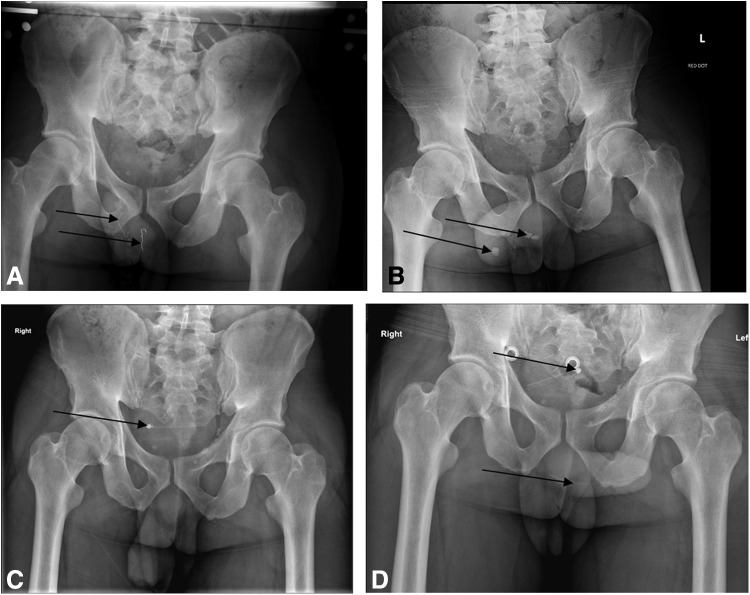
A selection of pelvic radiographs demonstrating FBs inserted into the patient's urethra, including parts of pens (**B**), paperclips (**A**), glass (**B**), and dismantled suprapubic catheter (**C, D**). FBs, foreign bodies.

This case describes the management of a 38-year-old Caucasian male prisoner, with a previous psychiatric history of borderline personality disorder and deliberate self-harm, who presented to the emergency department (ED) with a history of inserting multiple FBs into his urethra on 12 different occasions in the space of a 6-week period. Of these 12 attendances to the ED, 3 resulted in admission and 2 required transfer to the emergency operating theater for their removal under general anesthesia. Representative plain radiographs demonstrating the FBs are shown in Figure 1.

On one occasion, the patient developed acute urinary retention necessitating emergency insertion of a suprapubic catheter (SPC). Subsequently, the patient deliberately dismantled the SPC and reinserted it into the urethra. Thereafter, attendances to the ED became more frequent. After consultation with the prison and psychiatry services, it was decided that, whenever possible, hospital admission and urethral catheterization should be avoided. Further management plan at that stage was for a future elective admission for removal of the retained SPC tip and associated FBs from the bladder under general anesthesia.

In the subsequent week, the patient presented to the ED three times after insertion of further FBs. On each attendance, the FBs were easily palpable in the urethra and their removal was effective using forceps. However, on each occasion despite there being no other palpable FBs in the urethra, the patient was adamant that he was unable to void, until a catheter was inserted.

**FIG. 2.**
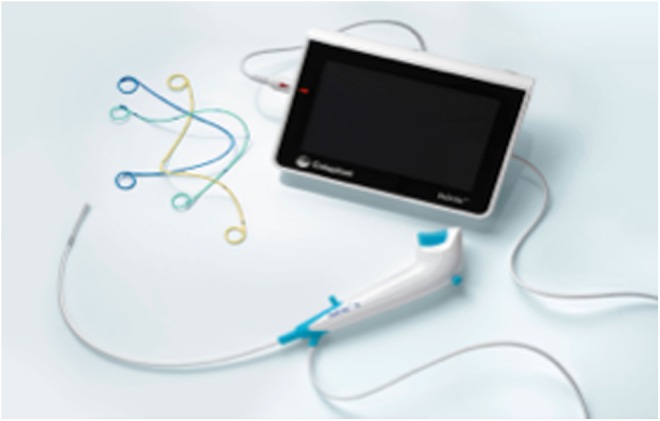
Showing the Isiris™ with monitor.

**FIG. 3.**
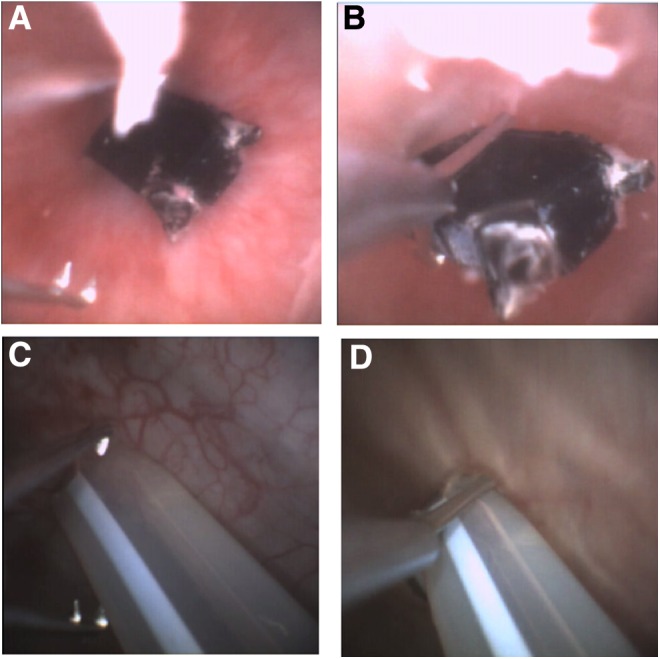
Still shots taken from the Isiris monitor while removing the FBs. **A, C** demonstrate how the stent grasping forceps are opened and **B, D** show how they are used to grasp and subsequently remove the FB.

**FIG. 4.**
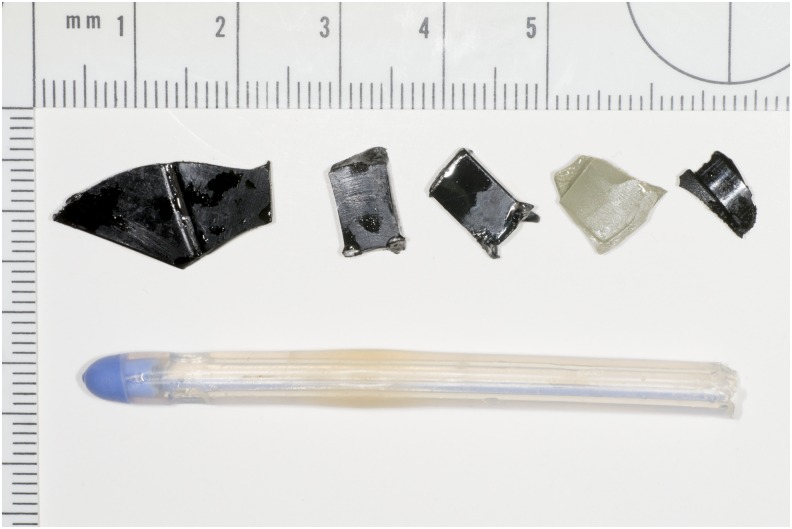
Various objects removed from the patient's urethra upon use of Isiris. Pictured are various parts of a dismantled pen and the tip of a dismantled catheter.

On his next attendance, the third in 5 days, it was decided to use Isiris™, a single-use flexible cystoscopy device with built-in stent grasper (Fig. 2), to remove the FBs and check the integrity of the urethra. The procedure was performed quickly within the ED, thus avoiding financial penalties for delay in discharge from the ED to a place of residence or to a hospital bed. The procedure only required intraurethral lubricant gel and two members of staff and it was well tolerated by the patient, only requiring basic oral analgesia. All remaining FBs that had been inserted over the previous 6 weeks were removed in this minimally invasive manner (See Figures 3 and 4). The procedure allowed a confident endoscopic examination to reassure the patient that no significant urethral trauma had been sustained, that there were no residual FBs, and that ultimately no reason for him not to be able to void. The use of the Isiris system permitted a safe and cost-efficient intervention with discharge directly from the ED.

## Discussion

Because of the rare nature of these cases and the added psychiatric component, a holistic approach to the management of polyembolokoilamania is required. A multidisciplinary approach was adopted with consultation of all key stakeholders (the patient, urology, psychiatry, ED, and prisoner services), resulting in a safe and efficient intervention. This minimized both reinforcement of the patient's behavior and the impact upon the organization's capacity and resources. Such a response was required because of the spiraling situation, culminating in three attendances in 5 days with the patient becoming more demanding and manipulative so as to remain an inpatient in hospital.

Although marketed as a stent removal device, Isiris enabled the removal of all the patient's FBs in one procedure in the ED. As Isiris is an all-in-one single-use system, there are multiple advantages of using it over rigid or flexible cystoscopy in such cases. First, the equipment can be kept in a 24-hour access storeroom and can be easily transported by a single person; it was taken from our storeroom to the patient and the procedure was performed in its entirety in the ED at the patient's bedside. This negated the need for admission of the patient and subsequent transfer to the emergency operating theater suite for conventional flexible or rigid cystoscopy.

A single-use Isiris cystoscope plus monitor costs £258 ($333). Table 1 illustrates a comparative cost breakdown for FB removal from the GU tract in both the ED and the emergency operating theater for organizations such as the UK's National Health Service (NHS). As Isiris is an all-in-one system that can be operated by a single individual, the cost savings are significant compared with FB removal in the operating theater with the need for an anesthetist, theater nurse, surgeon, assistant, operating department practitioner, and recovery staff; approximated at £1140 ($1470). Furthermore, as Isiris is a single-use system, the cost of cleaning the theater, sterilizing equipment, and disinfecting cystoscopes is also avoided.

**Table 1.** Illustrating the Costs Involved in Different Stages of Removal of a Foreign Body from the Genitourinary Tract

	*Admission*	*ED using Isiris*™
ED attendance	£132 ($170)	£132 ($170)
24 hours on a ward	£303 ($391)	—
1 hour emergency theater	£1000 ($1291)	—
Equipment cost	£95 ($122)	£258 ($333)
Total	£1530 ($1975)	£390 ($503)

Costing's are based upon U.K. NHS practice.^2,3^

ED = emergency department; NHS = National Health Service.

Cost is not the only advantage of being able to remove FBs in the ED. There are a number of patient factors that would make immediate endoscopic removal of FBs in the ED favorable, in particular the ability to avoid a general anesthetic. The procedure itself is well tolerated and markedly reduces time in hospital, allowing a quicker return to normal activities. Furthermore, endoscopic removal of FBs using Isiris can be performed by a wide spectrum of clinicians who are competent to perform flexible cystoscopies, including more junior medical staff. This further reduces waiting times for patients. Removing the FBs immediately upon presentation may also offer the potential benefit of avoiding the complications associated with urethral polyembolokoilamania such as strictures, retention, periurethral abscess, and fistulation.

There are many reasons people insert objects into natural orifices and there is a vast amount of literature surrounding polyembolokoilamania and the psychologic nature of the disease. Malingering refers to the act of deliberately feigning physical or psychologic symptoms to achieve a tangible secondary gain. In this case, the patient had a substantial and lengthy history of nonsuicidal, self-injurious malingering behavior. The phenomenon of urethral polyembolokoilamania by prisoners in a maximum-security hospital to control prison and hospital staff has been reported before and is difficult to manage.^4^ By avoiding admission and dealing with the problem within the ED, there is the potential, along with the help of psychiatry/psychology, to begin to break these patterns of behavior and relieve some of the economic burden posed by malingering patients.

## Conclusions

Although this scenario is admittedly rare, it provides a novel way of dealing with urethral polyembolokoilamania. Isiris is an easy-to-use device with which a urology specialist nurse or urology resident would be familiar. We have reported its utility for efficiently and safely removing urethral FBs, even out of hours. It requires minimal staffing support and can be done in the ED itself, thereby providing better and more expedient patient care. It has the potential to reduce the likelihood of complications associated with urethral polyembolokoilamania, while also saving resources in addition to preserving the availability of the operating theater for other surgical emergencies.

## Abbreviations Used

FB: foreign body
ED: emergency department
SPC: suprapubic catheter
NHS: National Health Service
